# Markers of Oxidative Stress in Patients with Acne: A Literature Review

**DOI:** 10.3390/life13071433

**Published:** 2023-06-23

**Authors:** Gabriela Loredana Popa, Cristina Iulia Mitran, Madalina Irina Mitran, Mircea Tampa, Clara Matei, Mircea Ioan Popa, Simona Roxana Georgescu

**Affiliations:** 1 Department of Microbiology, Carol Davila University of Medicine and Pharmacy, 020021 Bucharest, Romania; gabriela.popa@umfcd.ro (G.L.P.); irina.mitran@drd.umfcd.ro (M.I.M.); mircea.ioan.popa@umfcd.ro (M.I.P.); 2 Department of Dermatology, Carol Davila University of Medicine and Pharmacy, 020021 Bucharest, Romania; clara.matei@umfcd.ro (C.M.); simona.georgescu@umfcd.ro (S.R.G.); 3 Department of Dermatology, Victor Babes Clinical Hospital for Infectious Diseases, 030303 Bucharest, Romania; 4 Department of Microbiology, “Cantacuzino” National Medico-Military Institute for Research and Development, 011233 Bucharest, Romania

**Keywords:** acne vulgaris, oxidative stress, markers, isotretinoin

## Abstract

Acne vulgaris is a chronic inflammatory skin disease of the pilosebaceous unit. Its pathogenesis is multifactorial and involves the overlap between four main processes: alteration of the keratinization, increased sebum production, colonization with *Cutibacterium acnes*, and inflammation. The role of oxidative stress (OS) has been intensively studied in inflammatory skin conditions such as psoriasis, vitiligo, or atopic dermatitis. However, the involvement of OS in the pathogenesis of acne is less known. The evidence accumulated over the last decade suggests that in the case of acne patients, there is an imbalance between oxidants and antioxidants. In this review, we analyzed studies that evaluated markers of OS in patients with acne, published in the last ten years, with the aim of providing new insights into the pathogenesis of acne.

## 1. Introduction

Acne vulgaris is a common inflammatory skin condition of the pilosebaceous unit that affects most individuals between 12 and 25 years of age [1]. It manifests in two forms: classic acne, which usually appears at the age of 14, and late-onset acne, which occurs around the age of 30. Although females are more prone to acne, males are more likely to develop severe forms [2].

The major pathogenic factors that contribute to the onset and progression of acne lesions are the alteration of the keratinization process, increased sebum production, colonization with *Cutibacterium acnes* (*C. acnes,* formerly: *Propionibacterium acnes*), and inflammation. However, it is worth noting that genetic factors also play a significant role [3,4,5]. Altered keratinization can lead to the formation of comedones, while changes in sebum composition such as a reduction in the amount of linoleic acid can cause hyperkeratinization [6,7]. Hormones, especially androgens, are powerful inductors of sebaceous gland secretion and modulate keratinocyte proliferation [1,2]. Excessive sebum production creates a favorable environment for bacterial growth, which, in turn, leads to inflammation [7]. 

The skin microbiome is a community of microorganisms that reside on the skin and plays a crucial role in maintaining skin homeostasis. *C. acnes* is a Gram-positive anaerobic microorganism that is part of the normal flora and contributes to the pathogenesis of acne [8]. Changes in the skin microbiome, which in most cases involve a decrease in microbial diversity and an increase in pathogenic bacteria, play a significant role in the appearance of acne lesions [9]. Although initially believed that *C. acnes* hyperproliferation is essential in the pathogenesis of acne, in fact, the severity of acne is associated with the loss of diversity of *C. acnes* phylotypes. Phylotype IA1 is dominant in patients with acne. The loss of *C. acnes* phylotype diversity acts as a promoter of immune system activation at the skin level. When a skin explant is incubated with only phylotype IA1, there is an increase in the expression of inflammation markers such as interleukin 6 (IL-6), IL-8, IL-10, and IL-17. This is in contrast to incubation with a combination of phylotypes IA1 + II + III, which does not lead to such upregulation [10]. It seems that *C. acnes* in acne lesions is more virulent than the strains isolated from normal skin. Acne-related strains have the ability to release porphyrins that lead to the formation of reactive oxygen species (ROS) and trigger an inflammatory process in keratinocytes, which is associated with an oxidant–antioxidant imbalance [10]. 

### 1.1. A Brief Summary of Oxidative Stress Markers

ROS have deleterious effects on cell components (protein-derived enzymes, lipid-rich membranes, nucleic acids, and carbohydrates), causing functional and structural alterations. Depending on the molecular targets of ROS action, OS markers show a wide variety. Therefore, they can be divided into four main classes: markers of lipid peroxidation, DNA oxidative damage, protein oxidation, and carbohydrate oxidation. The antioxidant molecules also represent an important source of biomarkers to evaluate OS [11,12]. Table 1 summarizes the main markers of OS used in clinical practice.

The most used markers to assess lipid peroxidation are malondialdehyde (MDA), 4-hydroxynonenal (4-HNE), and isoprostanes. MDA is a stable aldehyde originating from the breakdown of polyunsaturated fatty acids during the lipid peroxidation process [17,18]. It is a highly reactive compound and by interacting with proteins may alter their structure, resulting in neoepitopes, responsible for the initiation of an inflammatory process. Its toxic activity was linked to mutagenic effects [19]. Another aldehyde, 4-HNE, acts as a secondary messenger for OS, regulating various signaling pathways and gene expression [19]. Isoprostanes are compounds structurally similar to prostaglandins, but are generated independently of cyclooxygenase through the peroxidation of arachidonic acid by a non-enzymatic pathway. F2 isoprostanes represent the most significant group of isoprostanes [20].

One of the primary effects of OS is the oxidative damage of DNA; nuclear DNA is particularly vulnerable to OS. 8-Hydroxy-2′-deoxyguanosine (8-OHdG) is a crucial marker of DNA oxidation, and high levels have been detected in various types of tumors [21]. Another common marker of nucleic acid oxidation is 7,8-dihydro-8-oxoguanine (8oxoGuo), a product of RNA oxidation. 8oxoGuo promotes protein synthesis deregulation and the generation of structurally modified proteins [22]. 

Protein oxidation leads to thiol oxidation, aromatic hydroxylation, and carbonyl group formation [23]. Carbonyl groups are commonly used to detect protein oxidation, but cannot exactly indicate the source of OS [24]. Thiols are found in the structure of amino acids (e.g., cysteine) and proteins, and exert an antioxidant activity [25]. During periods of OS, thiols interact with prooxidant molecules, leading to their transformation into compounds that have reduced reactivity. Thiols enter the oxidation reaction and generate disulfides through the transfer of excess electrons from ROS to thiols. Disulfides participate in the augmentation of OS and weaken the antioxidant system [26]. Thiol–disulfide exchange reactions are crucial in cellular homeostasis. The transformation of thiols into disulfides is a reversible reaction [27,28]. To evaluate thiol disulfide homeostasis, specific markers are used including native thiol, total thiol, and disulfides [29]. In recent years, a new marker for protein oxidation, namely, ischemia-modified albumin (IMA), has been intensively studied. Albumin, the primary protein found in human plasma, is crucial for maintaining body homeostasis. Its deficiency has been linked to higher mortality rates and an increased risk of acute coronary heart disease. The albumin can bind certain metals such as cobalt, copper, and nickel to its amino terminal end. When the body undergoes ischemia or OS, the structure of the amino terminal end of albumin is altered. This altered version of albumin is known as IMA [30,31].

Advanced glycation end products (AGEs) are a diverse group of compounds generated by irreversible nonenzymatic reactions including carbohydrates, proteins, lipids, or nucleic acids [32]. Pentosidine, carboxymethyllysine, and methylglyoxal are the most widely known AGEs and are used as biomarkers [32]. Although they are produced continuously under physiological conditions, they do not accumulate due to receptor systems that bind and remove them. When high amounts of AGEs accumulate, they can cause various effects such as an alteration in vasoregulation, the accumulation of extracellular matrix, inflammation, and dysregulated expression of growth factors [33,34]. The receptor for advanced glycation end products (RAGE) is a transmembrane receptor expressed in small amounts in tissues, but its expression increases when its ligands accumulate. The interaction between AGEs and RAGE leads to the augmentation of OS and the development of an inflammatory process by interrupting normal intracellular signaling pathways [35]. 

Measuring the concentrations of oxidant species is difficult, time-consuming, and expensive, therefore, considering that the effects of oxidant molecules are additive, the determination of total oxidant status (TOS) is preferable [36]. Along the same line, due to the difficulty of measuring different antioxidant molecules individually and considering their cumulative antioxidant effects, the total antioxidant status (TAS) of a sample is measured. OSI levels are calculated using the following formula: OSI (arbitrary units) = TOS (µmol H2O2Eq/L)/TAS (µmol Trolox Eq/L) [37].

### 1.2. Oxidative Stress—A Result of Cutibacterium Acnes-Induced Skin Inflammation

Physiologically, antioxidant systems can prevent the generation of ROS at the level of the pilosebaceous unit. However, under OS conditions, the antioxidant systems are exceeded, resulting in the alteration of the function and molecular composition of keratinocytes and sebocytes [38]. Since the 1990s, the possible role of OS in the pathogenesis of acne has been suggested. Akamatsu et al. showed that ROS generated in neutrophils led to the destruction of the follicular wall, an event that contributes to the progression of the inflammatory process [39]. Under physiological conditions, the pilosebaceous unit is an unfavorable environment for the development of anaerobic bacteria. OS produces changes in the microenvironment, resulting in a micromedium conducive to colonization with these species. Moreover, the oxidation of sebum in the follicle alters the oxygen concentration [40]. Follicular hyperkeratinization has been described as one of the main mechanisms involved in the pathogenesis of acne. However, studies have shown that the proliferation rate of keratinocytes does not differ between hair follicles of a patient with acne and one without acne. The excess of sebum and the accumulation of keratinocytes at the hair follicle level can lead to its obstruction, creating favorable conditions for the proliferation of *C. acnes* [41]. All of these events contribute to the development of *C. acnes*.

*C. acnes* plays a defining role in the initiation of inflammation by releasing chemotactic factors for neutrophils, which leads to their accumulation in acne lesions. Neutrophils, in turn, release ROS, which cause tissue damage, a phenomenon called “auto-oxidative damage” [42]. It has been observed that the level of hydrogen peroxide produced by neutrophils in the peripheral blood is significantly higher in patients with inflammatory acne lesions compared to healthy individuals [43]. The first step in the development of inflammation is the disturbance of the follicular epithelium by *C. acnes*, which enables bacteria in the microcomedones to interact with different skin and immune cells such as keratinocytes and macrophages. In the anaerobic and sebum-rich conditions of the follicle, *C. acnes* can stimulate immune cells to release proinflammatory cytokines such as IL-1 alpha, IL-8, and tumor necrosis factor (TNF) alpha, contributing to the inflammatory response [44]. Moreover, Tsai et al. showed that *C. acnes* can promote the expression of inducible nitric oxide synthase (iNOS) and cyclooxygenase (COX)-2 in the presence of ROS, which lead to the stimulation of activator protein 1 (AP-1) and the nuclear factor kappa B (NF-kB) pathway in macrophages. It is known that elevated levels of nitric oxide and prostaglandins are associated with harmful effects in the human body such as neoplasms, neuronal degeneration, or sepsis [44]. 

Moreover, in sebocytes, *C. acnes* has the ability to activate the NLRP (nucleotide-binding domain and leucine-rich repeat protein) inflammasomes, which are multiprotein complexes involved in innate immunity and inflammation. NLRP3 inflammasome activation is a process that requires proteases and ROS. Following inflammasome activation, IL-1 beta and IL-18 are released via caspase-1 activity from sebocytes and exacerbate the inflammatory process. NLRP3-deficient mice show less inflammatory cytokine production compared with wild-type mice [45]. Additionally, *C. acnes* releases lipase, which acts on fatty acids in sebum, increasing the concentration of palmitic and oleic acid, resulting in the activation of the inflammasome [46].

The immune response in acne involves a complex interplay between the innate and adaptive immunity. *C. acnes* strains have the ability to modulate CD4 + T-cell responses in various ways. This modulation can result in the generation of T helper (Th)-17 cells, which may play a role in either maintaining balance in the human body (by producing IL-17/IL-10) or contributing to the development of acne (by producing IL-17/interferon (IFN)-gamma) [10]. Th17 cells are recognized as inducers of inflammation, with significant evidence coming from studies on patients with psoriasis. *C. acnes* has the ability to activate genes related to IL-17 and IL-22 in human peripheral blood mononuclear cells. Additionally, IL-17 positive cells have been identified in acne lesions. Vitamins A and D have been shown to block *C. acnes*-induced Th17 differentiation, emphasizing their role in acne therapy [47,48]. Lipopolysaccharides (LPS) in the structure of *C. acnes* induce toll-like receptor (TLR)-2 expression in monocytes, leading to the release of TNF-alpha, IL-1beta, and IL-8, attracting other immune cells. TLRs can be seen as inducers of ROS production in various immune cells such as dendritic cells and macrophages. Therefore, it has been hypothesized that OS and the inflammatory process in acne occur through TLR activation by different antigens of *C. acnes*, especially LPS [49]. Moreover, *C. acnes* induces the release of metalloproteinases from monocytes, enzymes that are involved in numerous signaling pathways [46]. 

The aim of our review was to analyze the results of studies that have evaluated markers of oxidative stress (OS), published in the last decade, in order to understand the role of OS in acne pathogenesis and to find reliable markers to assess the oxidative status in acne patients.

## 2. Materials and Methods

We conducted a narrative review using the PubMed and Google Scholar databases. The search strategy used the keywords “markers”, “oxidative stress”, and “acne”. We defined the inclusion criteria as original articles written in English and published within the last 10 years (March 2013–March 2023). We excluded reviews, abstracts, clinical cases as well as original articles in which the method and study design were not clearly defined.

## 3. Results

We identified 24 articles that we divided into two categories: 19 articles that evaluated markers of OS in patients with acne compared to a control group (Table 2), and five articles that evaluated markers of OS in patients with acne before and after treatment with isotretinoin (Table 3).

## 4. Discussion

OS represents an imbalance between prooxidant and antioxidant molecules, resulting in a prooxidant status. There are increasingly more studies showing the involvement of OS in disease pathogenesis [74]. Researchers have suggested a classification based on the role of OS in disease pathogenesis; diseases in which OS represents the primary cause (e.g., atherosclerosis) and diseases in which OS plays a secondary role, being involved in the progression of the disorder (e.g., chronic obstructive pulmonary disease). However, in many cases, the contribution of OS remains incompletely elucidated [75]. OS has begun to be studied in more and more dermatological diseases, the skin being a major target of OS [76,77,78]. In the skin, ROS alter signaling pathways, induce the release of pro-inflammatory molecules, and increase the expression of vascular endothelial growth factor in keratinocytes [79]. In the last decade, there have been many studies that clearly indicate that patients with acne experience increased cutaneous and systemic OS. 

### 4.1. Oxidative Stress Markers Evaluated in Acne Patients

TAS, TOS, and OSI are the main markers used to globally evaluate OS. The results of the studies analyzed in our review are contradictory. Serum TOS and TAS levels were higher, lower, or without significant variation in patients with acne compared to the control group. Only one study evaluated the link between the serum levels of TAS, TOS, and OSI and disease severity and duration. Acer et al. found significantly higher levels of TAS, TOS, and OSI in acne patients compared to healthy subjects but they did not find any significant correlations between the serum levels of these parameters and the duration or severity of acne lesions. It is worth noting that the study included patients with mild and moderate acne. The authors explained the elevated level of TAS as a response to OS. TOS has been significantly higher than TAS, therefore, the OSI was also higher in acne patients compared to the controls [50]. 

Lipid peroxidation was assessed by measuring the serum levels of MDA, except for one study where the level of lipid peroxides in sebum was determined. In all studies analyzed, the serum level of MDA was statistically significantly higher in patients with acne compared to the control group. Additionally, higher levels of lipid peroxides and oxidized squalene were identified in the sebum. Acne is known to be associated with increased sebum secretion and an increased amount of squalene. In the case of a deficient antioxidant system, the peroxidation of fatty acids in sebum occurs, leading to the activation of an inflammatory process [63]. Studies indicate that lipids are involved in triggering an inflammatory process in several skin diseases such as psoriasis vulgaris, lichen planus, etc. [80,81]. An adequate balance between lipid peroxidation products with pro-inflammatory effects and those with anti-inflammatory properties is essential for regulating the inflammatory process. It is known that MDA induces the release of pro-inflammatory cytokines [82,83]. However, there was no notable correlation between the lipid peroxidation indices in sebum, and the concentration of IL-1alpha in the stratum corneum obtained from both affected and unaffected skin [68]. Awad et al. did not observe a correlation between the serum levels of MDA, TAS, and zinc and disease severity [59]. Furthermore, Ibrahim et al. did not find a correlation between the level of lipid peroxidation assessed by plasma MDA and the activity of erythrocyte G6PD, plasma CAT, and erythrocyte SOD enzymes [63]. 

DNA oxidation was evaluated by the serum 8-OHdG levels, which were higher in acne patients compared to the control group. High amounts of 8-OHdG are often associated with a chronic inflammatory process. Interestingly, Korkmaz et al. found that the serum levels of 8-OHdG did not correlate with the TAS and TOS levels [60]. A positive correlation was observed between the level of 8-OHdG and acne severity in the studies conducted by Korkmaz et al. [60] and Hagag et al. [54]. However, El Hadidia et al. revealed no correlation between DNA damage and acne severity [62]. Of note, El Hadidia et al. compared patients with vitiligo to those with acne in terms of OS, as the imbalance between oxidants and antioxidants is well-known in vitiligo. They observed that the level of OS was similar in patients with severe acne and vitiligo, suggesting a possible role for antioxidant therapies [62].

Analyzing the studies included in this review, we observed that in order to assess the protein damage in acne patients, greater attention was given to IMA (three studies). Other parameters evaluated were thiol-disulfide homeostasis markers (one study) and the serum levels of SDMA and ADMA (one study). Initially, IMA was used as a marker of ischemia in cardiovascular diseases. However, increased levels have been identified in numerous conditions including dermatological disorders such as atopic dermatitis, psoriasis, vitiligo, etc. [84]. In all studies, the serum levels of IMA were statistically significantly higher in patients with acne compared to the control group. Gurel et al. divided the patients into three groups (patients with mild, moderate, and severe acne) and observed that there were no significant differences regarding the serum IMA level [56]. These results are consistent with those obtained by Ebrahim et al. [52].

The transformation of thiols into disulfides is a marker of protein oxidation. Recently, thiol-disulfide homeostasis has been investigated in several cutaneous inflammatory diseases [25]. Current evidence suggests that the balance between thiols and disulfides mirrors the balance between oxidants and antioxidants [85]. Gurel et al. performed the first study assessing thiol-disulfide homeostasis in acne and suggested that the OS seen in patients with acne is not related to disulfide levels [58]. Post-translational modifications of proteins are other significant events that take place under OS conditions. SDMA and ADMA are molecules produced through post-translational modifications from arginine. ADMA represents the primary inhibitor of nitric oxide (NO) synthase and SDMA indirectly inhibits NO synthesis by causing arginine deficiency [86,87]. SDMA and ADMA appear to play an important role in numerous biological processes including OS, inflammation, and endothelial dysfunction [88]. Under OS conditions, there is an increase in the activity of enzymes responsible for the generation of SDMA and ADMA as well as a decrease in the activity of enzymes involved in their metabolism [89]. The only study that assessed SDMA and ADMA levels in acne patients revealed higher serum levels of these compounds [55].

Antioxidants may increase the expression of redox transcription factors such as nuclear factor erythroid 2-related factor 2 (Nrf2), NF-kB, AP-1, and mitogen-activated protein kinases (MAPKs) [90]. A wide range of molecules with antioxidant activity, enzymes, vitamins, trace metals, and glutathione were evaluated in patients with acne. Most studies revealed low serum levels of antioxidants. Garem et al. showed that SOD was significantly lower in patients with severe acne compared to those with moderate or mild acne, suggesting that the antioxidant defense systems’ capacity is exceeded in the advanced stages of the disease [64]. Al-Shobaili et al. also identified significantly lower levels of the antioxidant enzymes SOD and CAT in patients with severe acne compared to those with moderate forms [66]. In addition, Ozuguz et al. highlighted a negative correlation between serum levels of vitamin A and zinc and acne severity [67]. Ibrahim et al. suggested that the reduction in G6PD activity in acne patients may influence antioxidant levels in the epidermis. G6PD is a rate-limiting enzyme in the pentose-phosphate pathway, which is responsible for the production of NADPH. Furthermore, the NADPHs generated by G6PD are crucial for synthesizing the reduced forms of glutathione and thioredoxin. These reduced forms of antioxidants play a vital role in regenerating and maintaining an optimal antioxidant status [63].

It is very difficult to measure ROS directly, therefore, the products resulting from the action of OS are measured. Most of the studies are carried out in vitro and aim to measure the products resulting from lipid peroxidation, protein oxidation, and DNA damage. Different techniques have been used including spectrophotometric method, colorimetric method, enzyme-linked immunosorbent assay (ELISA), high-performance liquid chromatography (HPLC), etc. The determination of OS markers is most commonly performed using blood, saliva, tissue, and urine samples [91]. In the studies analyzed in our review, blood samples were used, with the exception of one study in which the measurements were made in sebum. The diversity of the results obtained can be explained by the heterogeneity of the study participants (age, severity of acne) and the different methods used.

### 4.2. Oxidative Stress Markers Evaluated in Acne Patients Treated with Isotretinoin

Isotretinoin is a derivative of vitamin A and is the treatment of choice for moderate and severe acne. Orally administered isotretinoin inhibits the activity of the sebaceous glands and exerts an anti-inflammatory and immunomodulatory effect by downregulating TLR2 and TLR4 expression and reducing the activity of Th cells. It is associated with numerous adverse effects, which have represented an important barrier to its use [92]. Recent research indicates the link between isotretinoin and OS, isotretinoin participating in the imbalance between oxidants and antioxidants, a fact that can be involved in the adverse reactions produced by this drug.

Georgala et al. conducted the first study that demonstrated the influence of isotretinoin on OS. They showed, in a group of patients with cystic acne, that after 45 days of treatment with isotretinoin, the TAS serum level was lower, and the serum level of 8-OHdG was higher compared to the values before treatment initiation [93]. However, Demir et al. did not detect differences regarding the level of TAS before and after treatment with isotretinoin, but observed increased levels of TOS and OSI [94]. Erturan et al. suggested that adverse effects associated with the treatment with isotretinoin such as mucocutaneous lesions, changes in liver enzymes, or plasma lipids may be a consequence of increased levels of OS induced by the drug [95].

The studies analyzed in this review support the hypothesis of the occurrence of OS following isotretinoin therapy, a hypothesis that was stated almost two decades ago. Izmirli et al. showed that the serum levels of 8-OHdG and hOGG1, a biomarker for 8-OHdG repair, increased significantly after the treatment with isotretinoin and suggested that the serum levels of hOGG1 increased in response to DNA damage but was not sufficient for DNA repair [71]. However, Alaqrawi et al. did not find statistically significantly differences between the level of 8-OHdG before and after the treatment with isotretinoin [69].

In the study conducted by Akturk et al., the serum levels of vitamin E decreased after the treatment with isotretinoin. Three mechanisms were suggested that can explain the low levels of alpha-tocopherol as a result of isotretinoin action including accelerated turnover of alpha-tocopherol in both the blood and tissues, reduced absorption, or amplified degradation of the drug in the intestine [73]. Interesting results were also found in the study by Alaqrawi et al., who showed that the addition of vitamin C along with isotretinoin could improve OS [69].

The OS induced by isotretinoin may be involved in therapeutic failure. Al-Kathiri et al. reported a case of severe acne resistant to isotretinoin that had a favorable outcome following dapsone therapy. Dapsone inhibits myeloperoxidase activity in neutrophils, which blocks the generation of ROS [96]. Further studies are needed to clarify the role of OS in the therapeutic response to isotretinoin.

## 5. Conclusions

In patients with acne, the balance between oxidants and antioxidants is altered. The most commonly used markers to assess OS in acne were MDA, a marker of lipid peroxidation, and the antioxidant enzymes CAT and SOD, which were measured in blood samples (serum/plasma/erythrocytes). Studies that have evaluated correlations between serum levels of OS markers and disease severity are few and the results are not consistent. Isotretinoin can increase OS, an effect that could be countered by antioxidant therapies. However, further studies on larger patient groups are needed. OS may be an important player in the pathogenesis of acne and deciphering the pathogenic mechanisms responsible for OS may represent the theoretical basis for the development of new therapies.

## Figures and Tables

**Table 1:** Markers of oxidative stress [13,14,15,16].

Process	Marker
Lipid peroxidation	MDA, 4-HNE, TBARS, F2-isoprostanes, acrolein, advance lipid oxidation products
Nucleic acid oxidation	8-OHdG, 8oxoGuo, 8-nitroguanine
Protein oxidation	Carbonyl groups, SH-groups, 3-nitrotyrosine, 3-chlorotyrosine, IMA
Carbohydrate oxidation	AGEs

MDA—malondialdehyde; 4-HNE—4-hydroxynonenal; TBARS—thiobarbituric acid reactive substances; 8-OHdG—8-hydroxy-2′-deoxyguanosine; IMA—ischemia modified albumin; AGEs—advanced glycation end products.

**Table 2:** Oxidative stress markers in acne patients vs. controls.

Markers	Study Participants (Number and Age)	Biological Material	Acne Severity	Acne Patients vs. Controls	Conclusions	Reference
TOS, TAS, OSI	32 acne patients (18–25 years) 15 healthy subjects (18–25 years)	Serum	Mild/moderate	TAS—higher TOS—higher OSI—higher	OS can participate in the onset of acne lesions or it can be the result of this condition.	Acer et al. (2022) [50]
MDA, GSH, OS related miRNAs—miRNA-31, miRNA-200a, and miRNA-21	57 acne patients (22.4 ± 3.7 years) 40 healthy subjects (23.4 ± 1.5 years)	Plasma, serum	Severe	MDA—higher, GSH—lower, miRNA-21—higher miRNA-200a and miRNA-31—no significant difference	OS may be involved in acne pathogenesis and antioxidant therapy may have a role.	Calis et al. (2022) [51]
IMA, GSH-ST, CAT, MDA	30 acne patients, 18 healthy subjects	Serum	Mild/moderate/severe	IMA—higher GSH-ST—higher MDA—higher CAT—lower	IMA can represent a marker that independently predicts disease activity and susceptibility.	Ebrahim et al. (2020) [52]
Vitamin E	17 acne patients (15–19 years) 17 healthy subjects (16–19 years)	Serum	-	Vitamin E—lower	Low levels of vitamin E are involved in altering the oxidant–antioxidant balance at the level of the pilosebaceous unit.	Ratnaningtyas et al. (2020) [53]
8-OHdG	15 acne patients (19.21 ± 5.05 years) 75 healthy subjects (20.53 ± 7.18 years)	Serum	Mild/moderate/severe	8-OHdG—higher	OS in acne can be associated with DNA damage.	Hagag et al. (2020) [54]
ADMA, L-NMA, SDMA, IMA Citrulline Vitamin A, Vitamin E	90 acne patients (18.67 ± 3.36 years) 30 healthy subjects (19.7 ± 2.49 years)	Serum	Mild/moderate/severe	ADMA—higher L-NMA—higher SDMA—higher IMA—higher Citruline—lower Vitamin A—lower Vitamin E—no significant difference	IMA and L-arginine—NO pathway may be involved in the pathogenesis and progression of acne lesions.	Akyurek et al. (2020) [55]
IMA	74 acne patients (18.54 ± 3.40 years) 60 healthy subjects (17.68 ± 3.7 years)	Serum	Mild/moderate/severe	IMA—higher	IMA can be considered a marker of OS in acne patients.	Gurel et al. (2019) [56]
25 -hydroxyvitamin D, adiponectin, MDA, TAS	40 acne patients (22.48 ± 4.15 years) 40 healthy subjects (23.38 ± 4.16 years)	Serum	Moderate/severe	MDA—higher 25-Hydroxyvitamin D, adiponectin—no significant difference TAS—no significant difference	Lipid peroxidation is altered in acne patients.	Moazen et al. (2019) [57]
NT, TT, DS DS/NT, DS/TT, NT/TT	74 acne patients (18.54 ± 3.4 years) 60 healthy subjects (17.68 ± 3.37 years)	Serum	Mild/moderate/severe	NT—no significant difference TT—lower DS—no significant difference DS/NT—no significant difference DS/TT—lower NT/TT—no significant difference	The OS in acne vulgaris is caused by a mechanism that is not associated with the disulfide level.	Gurel et al. (2019) [58]
MDA, TAC, zinc	60 acne patients (22.13 ± 5.13 years) 40 healthy subjects (24.00 ± 6.81 years)	Serum	Mild/moderate/severe	MDA—higher TAC—lower, Zinc—lower	The level of OS is increased in patients with acne vulgaris, but does not correlate with the degree of anxiety or depression.	Awad et al. (2018) [59]
8-OHdG TAS, TOS, OSI	35 acne patients (20.77 ± 2.17 years) 30 healthy subjects (21.90 ± 2.99 years)	Serum	Mild/moderate	8-OHdG—higher TAS—lower TOS—lower OSI—higher	DNA damage occurs as early as the early stages of the disease.	Korkmaz et al. (2018) [60]
TAS, TOS, OSI	35 patients with acne and anxiety disorders (14.7 ± 1.9 years) 37 patients with acne, (15.1 ± 1.4 years) 35 healthy subjects (age-matched controls)	Serum	Moderate/severe	TAS, TOS, OSI—no significant difference	The oxidant–antioxidant balance in acne patients is not influenced by anxiety disorders.	Demirkaya et al. (2016) [61]
SOD, MDA, DNA damage	15 acne patients, (20.87 ± 4.55 years) 15 healthy subjects (18.93 ± 3.86 years)	Serum	Mild/moderate/severe	SOD—lower MDA—higher DNA damage—higher	OS is involved in acne and antioxidant therapies could play a role.	El Hadidia et al. (2016) [62]
SOD, CAT, MDA, G6PD	40 acne patients, (19.4 ± 3.1 years) 36 healthy subjects (aged-matched controls)	Erythrocyte/plasma	Mild/moderate/severe	SOD—lower CAT—lower G6PD—lower MDA—higher	There is an altered oxidant–antioxidant balance in patients with acne vulgaris.	Ibrahim et al. (2015) [63]
SOD, MDA	50 acne patients (20.2 ± 4 years) 20 healthy subjects (aged-matched controls)	Erythrocite/serum	Mild/moderate/severe	MDA—higher SOD—no significant difference	OS may be involved in the pathogenesis of acne.	Garem et al. (2014) [64]
SOD, MDA	27 acne patients (18.3 ± 3.1 years) 10 healthy subjects (aged-matched controls)	Serum	Mild/moderate/sever	MDA—higher SOD—higher	OS is an important player in the pathogenesis of acne.	Gaber et al. (2014) [65]
CAT, SOD, TAS, MDA	156 acne patients (20.22 ± 3.6 years) 46 healthy subjects (20.1 ± 3.3 years)	Plasma	Mild/moderate/severe	MDA—higher CAT, SOD—lower TAC—lower	Markers of OS in acne can be used to assess the severity of the disease and monitor the response to treatment.	Al-Shobaili et al. (2014) [66]
Vitamin A, vitamin E. zinc	94 acne patients (28.54 ± 8.30 years) 54 healthy subjects (30.45 ± 9.46 years)	Serum	Mild/moderate/severe	Vitamin A—lower Vitamin E—lower Zinc—lower	Diet could have a role in acne evolution, therefore products rich in vitamins A and E and zinc could have beneficial effects.	Ozuguz et al. (2014) [67]
lipid hydroperoxides, squalene and squalene oxidation products	18 acne patients (teenage patients) 10 healthy subjects (teenage patients)	Sebum	Mild	Lipid hydroperoxides and the percentage of oxidized squalene—higher	Oxidation of sebum lipids, in particular squalene, may be involved in the evolution of mild forms of acne into inflammatory lesions	Capitanio et al. (2014) [68]

MDA—malondialdehyde; 8-OHdG—8-hydroxy-2′-deoxyguanosine; NT—native thiol; TT—total thiol; DS—disulfide; GSH—glutathione; GSH-ST—glutathione synthetase; SOD—superoxide dismutase; CAT—catalase; GPX–glutathione peroxidase; TAS–total antioxidant status; TOS–total oxidant status; OSI—oxidative stress index; IMA—ischemia modified albumin; SDMA—symmetric dimethylarginine; ADMA—asymmetric dimethylarginine; LNMA—NG-Methyl-L-arginine; G6PD—glucose-6-phosphate dehydrogenase.

**Table 3:** Oxidative stress markers in acne patients before and after isotretinoin treatment.

Marker	Study Participants	Biological Material	Acne Severity	Before vs. After Isotretinoin	Conclusions	Reference
MDA, 8-OHdG	20 acne patients (15–43 years) treated with isotretinoin (before and after treatment (2 months) (group A) 20 acne patients (15–43 years) treated with isotretinoin and vitamin C (before and after treatment (2 months) (group B)	Plasma/serum	Moderate/ severe	Group A: MDA—higher 8-OHdG—no significant differences Group B: MDA, 8-OHdG—no significant differences	The treatment with isotretinoin is associated with lipid peroxidation and the addition of vitamin C may decrease this effect	Alaqrawi et al. (2023) [69]
Ferritin, ceruloplasmin, albumin, copper, uric acid, neutrophil to lymphocyte ratio	53 acne patients before and after treatment with isotretinoin (40 months)	Blood	Moderate/severe	Uric acid, and ferritin—higher Albumin, albumin/globulin ratio, copper, ceruloplasmin, and neutrophil/lymphocyte ratio—lower	Isotretinoin influences the level of OS in acne patients.	Hareedy et al. (2022) [70]
8-OHdG, hOGG1	43 patients (>18 years) before and after treatment with isotretinoin (6 months)	Serum	-	8-OHdG—higher hOGG1—higher	Isotretinoin treatment produces DNA damage.	Izmirli et al. (2020) [71]
PON1, TOS, TAS, OSI	35 acne patients (21.33 ± 3.36 years) before and after treatment with isotretinoin (3 months)	Serum	Severe	PON1—lower TOS—higher OSI—higher TAS—no significant differences	Alteration of the antioxidant–oxidant balance may be involved in the adverse events associated with isotretinoin treatment	Okzol et al. (2015) [72]
Vitamin E	46 patients (21.41 ± 4.15 years) before and after treatment with isotretinoin (5 to 7 months)	Serum	Moderate/ severe	Vitamin E—lower	Isotretinoin treatment is associated with a decreased serum levels of vitamin E.	Akturk et al. (2013) [73]

MDA—malondialdehyde; 8-OHdG—8-hydroxy-2′-deoxyguanosine; hOGG1—8-oxoguanine DNA N-glycosylase 1; PON1—paroxonase 1; TAS—total antioxidant status; TOS—total oxidant status; OSI—oxidative stress index.

## Data Availability

Not Applicable.
